# Strategies and Approaches for Discovery of Small Molecule Disruptors of Biofilm Physiology

**DOI:** 10.3390/molecules26154582

**Published:** 2021-07-29

**Authors:** Michael A. Trebino, Rahul D. Shingare, John B. MacMillan, Fitnat H. Yildiz

**Affiliations:** 1 Department of Microbiology and Environmental Toxicology, University of California, Santa Cruz, CA 95064, USA; mtrebino@ucsc.edu; 2 Department of Chemistry and Biochemistry, University of California, Santa Cruz, CA 95064, USA; rshingar@ucsc.edu

**Keywords:** biofilms, biofilm inhibitors, c-di-GMP inhibitors, inhibitor discovery, structure-activity relationship

## Abstract

Biofilms, the predominant growth mode of microorganisms, pose a significant risk to human health. The protective biofilm matrix, typically composed of exopolysaccharides, proteins, nucleic acids, and lipids, combined with biofilm-grown bacteria’s heterogenous physiology, leads to enhanced fitness and tolerance to traditional methods for treatment. There is a need to identify biofilm inhibitors using diverse approaches and targeting different stages of biofilm formation. This review discusses discovery strategies that successfully identified a wide range of inhibitors and the processes used to characterize their inhibition mechanism and further improvement. Additionally, we examine the structure–activity relationship (SAR) for some of these inhibitors to optimize inhibitor activity.

## 1. Introduction

Biofilms—microbial communities composed of microorganisms encased in an extracellular matrix—are the most abundant microbial growth mode on Earth [1]. Biofilms can have both beneficial and detrimental effects in environmental, industrial, and clinical settings. The ability to form biofilms is essential for the pathogenicity of several pathogenic bacteria. Biofilm-related infections are often associated with chronic diseases, and biofilms affect the virulence of acute infections and treatments’ efficacy [2,3,4,5]. When grown as biofilms, pathogenic microorganisms are recalcitrant to immune system clearance, inherently resistant to traditional antibiotics, rendering conventional antibiotics and therapeutics non-viable [6,7,8,9]. Biofilm formation represents another challenge in clinical settings, as pathogenic bacteria can form biofilms on implantable medical devices, resulting in device failure and chronic infections. Biofilms are involved in 65 to 80% of nosocomial infections [10,11,12,13]. There are no clinical antimicrobial agents currently existing that target biofilms; hence, the disruption of biofilm formation represents a valuable new target for antimicrobial discovery [14]. Furthermore, around the world, antibiotic resistance is increasing, and traditional treatment and prevention methods are becoming increasingly ineffective [15,16]. We need to identify and target different cellular processes for the development of new antimicrobial agents. 

In this review, we discuss inhibitor discovery methods and strategies and approaches for characterizing novel inhibitors. We will first provide an overview of biofilm formation, then discuss successful strategies in discovering and developing biofilm inhibitors, the mechanism of action and structure–activity relationships (SAR) for well-characterized inhibitors, and the broad classification of antibiofilm compounds and shared characteristics. We primarily focused on compounds whose mode of biofilm inhibition occurred by altering biofilm regulation and biofilm-forming ability rather than antimicrobial activity. Finally, we also discuss the growing topic of therapeutic drug-conjugates, which improve cell/biofilm penetration and the delivery of antibiotics to eradicate biofilms through antimicrobial activity. While the inhibitors discussed are not a comprehensive list, they represent a wide range of diverse biofilm inhibitors with differing methods of discovery and inhibitory targets. As there is a growing interest in biofilms as antimicrobial targets, additional biofilm inhibitors and their known action mechanisms have been discussed in recent reviews, including synthetic biofilm inhibitors such as the phenazine and amino-immidozole inhibitor classes [17,18,19,20,21,22,23].

## 2. Biofilm Formation and Targets for Anti-Biofilm Interventions

In totality, biofilm formation is a series of events needed for the transition from the planktonic lifestyle to a surface-associated multicellular community. Biofilm formation begins with surface attachment using various cell-surface appendages such as pili, flagella, and surface proteins [24,25,26,27,28,29,30,31,32]. The initial stage of biofilm formation is crucial, as the inhibition of surface attachment prevents surface colonization and circumvents the traditional advantages of the biofilm lifestyle. Following surface attachment, some bacteria move along the surfaces using different surface motilities, while others do not exhibit surface motility [33,34,35]. Surface-attached founder cells undergo cell-division and retain their progeny by producing an extracellular matrix resulting in microcolony formation and eventually mature biofilms [24,25,26,27,28,29,30,31,32,33,34]. Composed of exopolysaccharides, matrix proteins, and extracellular DNA, the biofilm matrix aids in cell–cell and cell-surface adherence, structural integrity, and results in emergent biofilm properties such as resistance to physicochemical stresses, including resistance to antimicrobial agents [30,35,36,37,38,39,40,41,42,43,44,45,46,47,48,49,50,51]. This resistance is due to multiple factors, including altered physiology of biofilm-grown cells, chemical inactivation of antimicrobials by the extracellular matrix, and decreased penetration of antibiotics within the biofilm [52,53]. As intact biofilms are difficult to eradicate, there is an interest in identifying compounds that prevent biofilm formation by targeting biofilm matrix production and its assembly.

**Figure 1:**
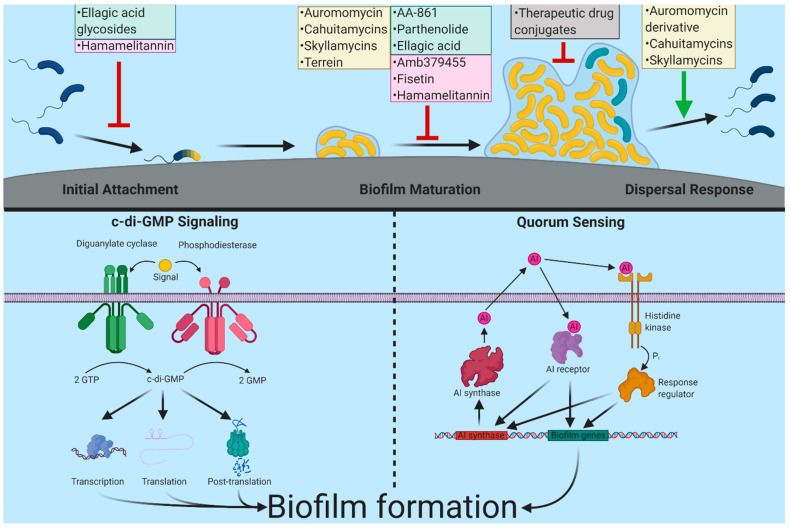
Biofilm inhibitors and their targets discussed in this review. Biofilm formation starts when planktonic cells (colored blue) sense and attach to the surface. Following this, surface-attached cells (colored yellow) begin to divide, recruit nearby cells, and produce biofilm matrix components and form microcolonies and eventually mature biofilms. In response to cellular signals or external cues, biofilms can disperse, and dispersed cells can colonize new environments. Major inhibitors covered in this review, as well as their discovery method, are shown by color. High throughput discovery is represented by yellow, targeted/pathway discovery by green, in silico discovery by pink, and therapeutic drug conjugates in grey. A schematic of two major regulatory systems governing biofilm formation c-di-GMP signaling and quorum sensing (QS) are indicated. C-di-GMP is produced by diguanylate cyclases (DGCs) and degraded by phosphodiesterases (PDEs). Signals can impact the enzymatic activity of these enzymes allowing for control over the intracellular concentrations of c-di-GMP. These changes in c-di-GMP can be sensed by receptors that affect transcription, translation, and activity or stability of biofilm-associated genes/proteins. Bacteria use quorum sensing to determine cell density. These systems produce an autoinducer compound, which is secreted into the environment. QS signals are then sensed by periplasmic proteins, membrane-bound histidine kinases or cytosolic receptors and initiate signal transduction pathways regulating biofilm gene expression. Created with BioRender.com.

Canonical regulatory circuitries controlling biofilm formation are another target of interest, as the inhibition of such circuitries can prevent biofilm formation. The regulation of biofilm formation is complex and involves several signaling molecules and regulatory circuitries. Nucleotide-based intracellular signaling molecule cyclic di-guanosine monophosphate (c-di-GMP), a broadly conserved bacterial signaling molecule, is the central regulator of biofilm formation [54,55,56,57]. C-di-GMP is produced by diguanylate cyclases (DGCs) and is degraded by phosphodiesterases (PDEs) [54,55,56,57]. C-di-GMP is sensed by receptor proteins or by c-di-GMP riboswitches to control cellular processes through transcriptional, post-transcriptional, and translational mechanisms that govern all stages of biofilm formation, including surface attachment, surface motility, and matrix formation [54,55,56,57,58]. Modulation of c-di-GMP production, c-di-GMP receptor function, and their cognate cellular targets are, therefore, one of the most crucial targets for developing anti-biofilm interventions. Other regulatory networks such as those involved in sensing cell density by autoinducers, i.e., quorum sensing (QS), or those involved in sensing and responding to environmental conditions, i.e., two-component regulatory systems (TCS), also govern the regulation of biofilm formation [59,60]. Compounds that inhibit these regulatory networks are also of interest and are widely explored [60,61,62,63] (Figure 1).

## 3. Approaches to Discover Anti-Biofilm Compounds

**Figure 2:**
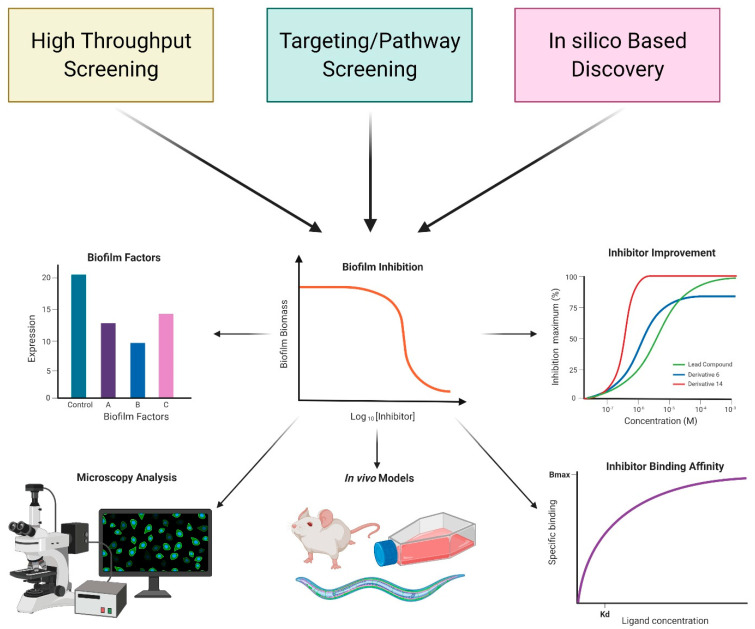
Overview of approaches used identification of biofilm inhibitors. Screening-based and in silico discovery approaches are commonly used. After initial discovery, typically using biofilm biomass or biofilm gene expression as a readout, additional studies are performed to determine the impact of these inhibitors on biofilm matrix production, biofilm structure, biofilm driven infection, inhibitor binding affinity, and inhibitor improvement. Such studies provide an insight into the mechanism of action of the identified compounds. Created with BioRender.com.

Biofilm inhibitors have been identified using various screening, high-throughput (HTS) and targeted/pathway screening, and in silico analysis approaches. Each of these strategies has its unique advantages and disadvantages, enhancing inhibitor discovery attempts. Upon discovery, potential inhibitors are tested for antibacterial activity, which is determined by measuring biomass or metabolic activity using methods evaluating cell density, cell counts, or ATP levels. Analysis of antibacterial activity is important in determining if a compound inhibits biofilm formation by modulating a biofilm-specific process or by cell viability (Figure 2).

One major group of biofilm inhibitors, inspired by scaffolds found in natural products with reported activity, are a diverse range of heterocyclic containing families, which include indoles [64,65,66,67,68,69,70,71], 2-aminoimidazoles [72,73,74,75,76,77,78,79,80,81,82,83,84,85,86,87,88,89], phenazines [90,91,92,93,94,95,96,97,98], and quinolines [99,100,101,102,103,104,105,106]. These lead molecules are the results of significant medicinal chemistry effort to improve potency and other drug-like properties. A prominent example of such studies is the work done by C. Melander and colleagues, where multiple biofilm inhibitors were developed based on a 2-aminoimidazole core inspired by the natural product bromoageliferin. These families of heterocyclic biofilm inhibitors have recently been extensively reviewed and will not be covered in this review [107,108,109,110].

In this review, we will focus on screening and in silico discovery efforts on selected pathogenic bacteria: *Acinetobacter baumannii*, *Vibrio cholerae*, *Pseudomonas aeruginosa*, *Escherichia coli*, and *Staphylococcus aureus*. 

## 4. Inhibitors Identified via Cell-Based or In Vitro Screens

**Figure 3:**
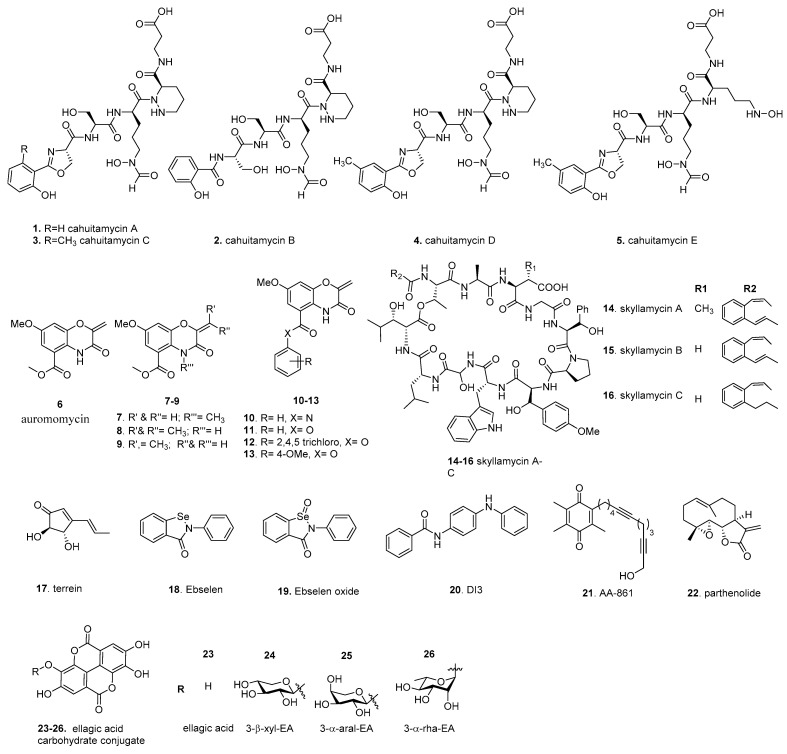
Screening-based discovery of biofilm inhibitors.

Unbiased screens, typically using biofilm biomass measurements as a readout, are often used to identify compounds impacting biofilm formation from natural products or synthetic chemical libraries. Targeted/pathway screens utilize specific information on biofilm formation, such as biofilm gene expression, enzymatic activity, along with a screening of natural products or synthetic chemical libraries to identify compounds that impact particular systems or pathways. In the following section, we discuss a set of biofilm inhibitors identified using these approaches (Figure 3). These compounds, along with those discussed in later sections, will have their published inhibitory and disruptive activity listed, including their target and discovery method for ease of comparison (Table 1).

### 4.1. Cahuitamycins

A widely utilized method for unbiased screening is biofilm staining, using crystal violet, to measure biofilm biomass [111,112]. In one such example, a library of 9831 marine microbial-derived extracts were screened to identify compounds that were able to inhibit *A. baumannii* biofilm formation. An extract from *Streptomyces gandocaennsis* was found to inhibit biofilm formation without impacting cell growth [113]. Subsequent studies identified the secondary metabolites cahuitamycins A–C (**1**–**3**) as the lead compounds (Figure 3). **3** had the most potent inhibitory potential of the three compounds with a half-maximal biofilm inhibitor concentration (BIC_50_) of 14.5 µM. Later, mutasynthetic studies, utilizing ribosomal engineering, led to the production of additional derivatives referred to as cahuitamycins D,E (**4**, **5**), which improved the BIC_50_ to 8.54 and 10.5 µM, respectively [114]. Of the biofilm inhibiting compounds, Park et al. reported a minimal impact on cell viability. Further studies of the biosynthetic pathway of the cahuitamycins found that CahJ, an adenylation enzyme, was important in cahuitamycin diversification, and due to CahJ substrate promiscuity, could be used to generate further derivatives for evaluation as potent biofilm inhibitors [115]. These new compounds also gained the ability to disperse preformed biofilms at a relatively high concentration. The above biofilm inhibitory activity of cahuitamycins suggested that the terminal 2-hydroxybenzoyl-oxazoline group represents a key pharmacophore. As the cahuitamycins have siderophore-like properties, the authors tested the iron-complexed form of **1**, which showed minimal biofilm inhibition. Interestingly, the loss of inhibitory activity over time occurred as a result of metal-complexed cahuitamycins forming. While the mechanism of action is currently unknown, Park et al. noted that the cahuitamycins impacted biofilm maturation and not initial attachment. These observations, including the gain of function for biofilm dispersal, suggests that this class of inhibitors primarily impacts biofilm maturation and integrity. 

### 4.2. Auromomycin

In the past decade, high content screening has gained favor as a whole-cell approach. It provides direct measurements of the impact on biofilm formation, such as altered architecture or maturation dynamics. To identify biofilm inhibitors in *V. cholerae*, Peach et al. used fluorescently tagged *V. cholerae* rugose variant, which has enhanced biofilm-forming ability due to high c-di-GMP production, in a biofilm image-based screen. A unique marine microbial natural products library from 1248 unique prefractions was screened; the central chromophore of auromomycin (**6**) exhibited the most significant degree of biofilm inhibition among the lead compounds [116]. (Figure 3) We note that auromomycin has been studied previously as an antitumor natural product that prevented the growth of lymphoblastoma L5178Y cells and that auromomycin also showed antimicrobial activity against Gram-positive and Gram-negative cells [117,118]. The impact of **6** on biofilm formation was further investigated using confocal scanning laser microscopy (CSLM) to understand its effects on biofilm formation. In a dose-dependent manner, **6** altered the appearance of mature biofilm architecture and integrity and reduced the size of microcolonies with a BIC_50_ of 60.1 µM. Interestingly, Peach et al. found that **6** did not impact the cell growth of *V. cholerae* or the cell viability of HeLa cells at 250 µM. As **6** is structurally identical to an alkaline degradation product of the auromomycin chromophore, the antimicrobial and antitumor activity likely requires the intact chromophore [119]. It was shown that **6** is unable to disperse preformed biofilms [120], suggesting that **6** acts primarily against the early stages of biofilm formation. **6** is a structurally new class of biofilm inhibitor comprising a benzo[1,4]oxazines core with an exocyclic olefin and does not have cytotoxicity at BIC_50_ concentrations, making it a desirable inhibitor candidate. 

A subsequent study focused on structural characterization and improvement of the auromomycin scaffold. Warner et al. reported the synthesis of **6**, along with a series of structurally simplified analogs for SAR studies [121]. The library of 41 simplified analogs was examined for anti-biofilm activity against *V. cholerae* biofilms in relation to structural modifications. It was discovered that the removal of the exocyclic double bond or adding substituents (**8**,**9**) to the double bond was detrimental to activity. This is highlighted by the α, β unsaturated carbonyl that acts as a Michael acceptor with potential involvement in the mechanism of action. Similarly, the N-methyl analog **7** was also found to be inactive, suggesting that the hydrogen bond donor is required for its activity in the active site. The modification of substituents on the ester **11**–**13** resulted in an increase in biofilm inhibition, whereas its amide counterpart **10** was completely inactive. The lead compound **13** displayed strong biofilm dispersal activity and no bactericidal activity, with a biofilm dispersal concentration (BDC_50_) value of 13 µM, a BIC_50_ of 6 µM and no mammalian cell cytotoxicity against HeLa cells up to up to 200 µM. Compound **13** was capable of disrupting *V. cholerae* biofilms under both static and flow cell conditions, resulting in a seven-fold reduction in biofilm biomass. Additionally, co-dosing of compound **13** with 50 µM erythromycin or azithromycin showed enhanced detachment and subsequent clearance of preformed biofilms, suggesting a synergistic action of a dispersal agent such as **13** and antibiotics. SAR studies, such as that above, are powerful tools in the characterization and improvement of novel inhibitors.

### 4.3. Skyllamycins

Building upon the success of auromomycin’s discovery, Navarro et al. performed a modified version of the biofilm image-based screen against a fluorescently tagged *P. aeruginosa* strain [122]. For this screen, a biofilm image-based screen was coupled with an XTT ((2,3-Bis-(2-methoxy-4-nitro-5-sulfophenyl)-2H-tetrazolium-5-carboxanilide) assay, which measures cellular metabolic activity via the reduction of XTT. Thus, this screen evaluates the impact of a compound on both biofilm formation and cell viability. The compounds were then sorted into quadrant-based bins based on biofilm inhibition and cellular activity to identify non-antibiotic activity inhibitors. Subsequently, two versions of this screen were used to model the modes of inhibitory action: the biofilm inhibition model (BIM) and the biofilm dispersal model (BDM). Such analysis provides insights into mechanisms of action. It allows the classification of compounds based on their ability to prevent biofilm formation via BIM and their ability to clear preformed biofilms via BDM. The authors focused on compounds corresponding to extracts with low biofilm coverage but with relatively normal cellular activity. They identified an extract containing skyllamycins A–C (**14**–**16**) as the most desirable compounds for biofilm inhibition and dispersal (Figure 3). **15** and **16** were found to have 50% effective concentrations (EC_50_), a term synonymous with BIC_50_, of 30 and 60 µM, respectively, where **14** had a relatively high EC_50_ of >250 µM. In all instances tested, **14**–**16** did not reduce cell activity, indicating that these compounds do not inhibit biofilms through antibiotic-mediated action. While **14** and **16** were unable to function as dispersal agents, **15** could clear preformed biofilms with an EC_50_ of 60 µM. The skyllamycins belong to a family of non-ribosomal cyclic depsipeptide natural products containing the very rare α-OH-glycine and the added functionality of three β-OH amino acids. Recent developments have led to the successful synthesis of skyllamycins opening the possibility of additional derivative studies for further inhibitory improvement [123]. As the skyllamycins are structurally distinct from quorum sensing mimics, these compounds likely act on a non-quorum sensing pathway, representing the first known class of cyclic depsipeptide biofilm inhibitors/dispersers. Co-dosing experiments of **15** and azithromycin, an antibiotic unable to clear pre-attached biofilms, demonstrated that, in combination, these compounds were able to eliminate surface-associated biofilms and lower cellular metabolic activity. A further study developed a solid phase peptide synthesis protocol and solution phase macrolactamization strategy to access the skyllamycins and four simplified analogs [124]. Interestingly, **14**–**16** and their deshydroxy analogs are found to have moderate biofilm inhibitory activity, suggesting that these variations are well-tolerated.

### 4.4. Terrein

Screening methodologies can be targeted towards particular aspects of biofilm formation [125]. This bias provides important contextual clues about the mechanism of inhibition. An example of this approach focused on an extracellular elastase activity, an important biofilm factor for late-stage biofilm maintenance and biomass accumulation in P. *aeruginosa*, as a readout of biofilm formation [126,127]. By using an elastin-Congo red conjugate, elastase activity can be measured as a function of increasing absorbance as the Congo red dye is released. From a microbial extract library of 12,300 compounds, terrein, (**17**) a compound isolated from the fungus *Aspergillus terreus*, was found to decrease elastase activity by 29.1% and 81.1% at concentrations of 30 and 100 µM, respectively, and could reduce biofilm biomass at those concentrations (Figure 3). Discovered in 1935, **17** has documented anti-cancer activity and minor antimicrobial activity; however, these effects were demonstrated at concentrations higher than those tested by Kim et al., suggesting that lower concentrations may allow for biofilm inhibition with minimal antimicrobial action [128,129,130]. **17** decreased elastase production, biofilm matrix production, and biofilm thickness. It was shown that the hydroxyl moieties on terrein are essential for such a decrease. Since **17** is structurally similar to the QS molecule acyl homeserine lactone (AHL), an AHL-based in vitro quorum sensing competition assay was utilized to determine whether terrein can block AHL receptors using homologs of the *P. aeruginosa* QS receptors LasR and RhlR. From this, it was found that **17** antagonistically inhibited both QS receptors’ ability to bind to their respective AHL compounds and subsequently inhibited QS-regulated genes. As QS and c-di-GMP signaling regulatory circuits regulate each other, exposure to terrein negatively impacted biofilm matrix production, decreased intracellular c-di-GMP, and attenuated virulence in *Caenorhabditis elegans* and the murine airway infection model [76].

### 4.5. Ebselen

C-di-GMP is a central regulator of biofilm formation; hence, inhibitors targeting the activity of c-di-GMP metabolizing enzymes and c-di-GMP receptors are of great interest. A recent study used a targeted c-di-GMP receptor and the differential radial capillary action of ligand assay (DRaCALA), which determines the fraction of the ligand bound to a specific protein, to identify competitive inhibitors [131]. This method was utilized to screen the NIH clinical collection 1 library for compounds that could reduce the fraction of bound ^32^P-c-di-GMP to PelD, a *P. aeruginosa* c-di-GMP receptor required for the synthesis of the exopolysaccharide Pel [132,133]. Ebselen (**18**), an organoselenium compound with drug-like properties and antioxidant, anti-inflammatory, and cytoprotective activity, was the only compound found to reduce c-di-GMP binding beyond 3 standard deviations of the positive cutoff. (Figure 3) Commercially purchased **18** and its oxide variant, the selenone analog (**19**), was able to reduce 32P-c-di-GMP binding to PelD and the *P. aeruginosa* DGC WspR by 80% and 90%, respectively, although this was not the case for PilZ domain c-di-GMP receptors or PDEs. Furthermore, **18** supplementation altered c-di-GMP dependent phenotypes, such as the motility and biofilm formation, while not impacting *P. aeruginosa* growth. Based on previous observations of ebselen modifying cysteine residues, Lieberman et al. proposed that **18** covalently modifies cysteine residues in the active site of DGCs. WspR has two cysteines at positions 49 and 240, one of which is adjacent to the RxxD I-site, which is important for c-di-GMP allosteric regulation of the catalytic domain [134].

### 4.6. DI-3

In another study targeting c-di-GMP metabolizing enzymes, Sambanthamoorthy et al. utilized a c-di-GMP inducible promoter, the upstream region of the *V. cholerae* gene VC1673, fused to a luciferase operon as a transcriptional reporter to identify compounds that decrease luminescence as a function of decreased c-di-GMP levels [135]. Over 66,000 unique compounds and natural product extracts were screened for a reduction in luminescence, which is also an indirect readout of decreased cellular-di-GMP levels. This study identified 184 compounds as being highly effective in reducing luminescence. Subsequently, Sambanthamoorthy et al. tested these compounds for their ability to inhibit the activity of a select group of purified DGCs in an in vitro analysis and impact on cell growth. The lead compound DI-3 (**20**) was found to inhibit the *V. cholerae* DGC VC2370 and the *P. aeruginosa* DGC WspR in a dose-dependent manner with in vitro IC_50_ values of 1.0 µM and 17.83 µM, respectively, without negatively impacting cell growth. (Figure 3) **20** is a linear molecule containing a biphenyl amine and one amide bond functional group and is in compliance with the Lipinski rule of five. All the lead compounds from this screen are linear and have a similar steric bulk, and 13 to 15 have the longest countable atomic linkages end to end. The presence of aromatic rings at both ends of the inhibitor suggests the folding of the molecule in half to achieve pi-pi stacking. This mimics pi-pi stacking of c-di-GMP to form higher-order multimers, and these multimers are required for binding to RXXD allosteric sites. Supplementation of **20** was able to reduce *V. cholerae* biofilm biomass, which was attributed to a decrease in c-di-GMP, as measured by mass spectrometry quantification. Using quantitative crystal violet staining on minimum-biofilm-eliminating concentration (MBEC) plates, the BIC_50_ for *V. cholerae* was found to be 26.2 µM.

### 4.7. AA-861 and Parthenolide

Bacteria with increased ability to produce biofilm matrix components form biofilms with distinct corrugated patterns resulting from matrix production and assembly [39,136,137,138]. This readily screenable phenotype of biofilm formation is used to identify biofilm inhibitors that can visibly impact these phenotypes. In one such example, a collection of bioactive compounds were screened for their impact on *B. subtilis* ability to form corrugated biofilms at the air–liquid interface, also known as pellicles [139]. The biofilm matrix of *B. subtilis* is composed primarily of exopolysaccharides (eps) and amyloid-like fibers formed from the protein TasA. This approach’s advantage is that wrinkles are a distinguishable critical feature and can be used to screen for molecules with anti-EPS and/or anti-amyloid activity. From the 480 compound BIOMOL–ICCB collection, two compounds were identified as having strong biofilm inhibitory activity and no impact on cell growth: AA-861 (**21**), a benzoquinone derivative with anti-inflammatory activity, and parthenolide (**22**), a germacrane sesquiterpene lactone obtained from the feverfew plant (Figure 3). By analyzing biofilm inhibition in the absence of one of the two major biofilm components, it was found that **21** and **22** inhibited biofilm formation via TasA. Further investigation of in vitro TasA polymerization, via a thioflavin T in vitro assay (fluorescence increases as thioflavin T binds to the β sheet-rich fold found in amyloids), showed that supplementation of either **21** or **22** at 50 µM to TasA led to a drastic reduction in fluorescence signal accumulation, indicating that these compounds inhibited TasA polymerization.

Furthermore, **21** and **22** had an additive effect in preventing TasA from forming amyloid-like fibers and could reduce biofilm biomass for *B. cereus* and *E. coli*. Though the mode of inhibition in those species is unclear, *E. coli* produces Curli, an amyloid fiber, and *B. cereus* has a homolog of TasA [48,139]. These findings suggest that these compounds may function as broad inhibitors of amyloid polymerization. Supplement of 100 µM of **22** was also able to disrupt preformed pellicles, indicating that it has dispersal qualities in addition to inhibition. The mechanism of action by which **21** and **22** reduce amyloidogenesis largely remains unclear. However, Romero et al. proposed that direct interaction of the inhibitors with different forms of the amyloid proteins could hamper the polymerization of the fiber. 

Besides impacting amyloid-like fiber assembly, **22** affected the expression of quorum sensing systems and reduced the production of biofilm matrix components for *P. aeruginosa* [140]. Additionally, in silico analysis showed that **22** has a strong potential of binding to the ligand-binding domain of LasR, offering a potential explanation for biofilm inhibition in *P. aeruginosa*. **22** may be a useful inhibitor, as it can inhibit biofilm formation among a wide range of bacteria and through different mechanisms and targets.

### 4.8. Ellagic Acid Carbohydrate Conjugates

One of the earlier examples of biofilm inhibitors is ellagic acid (**23**), a plant-derived compound [141]. (Figure 3) **23** and other polyphenolic compounds with a gallic acid moiety were shown to inhibit biofilm formation when investigating plant extracts for anti-quorum sensing activity. The root extract of *Rubus ulmifolius Schott*, a wild shrub from the Mediterranean used in traditional medicine, specifically the fraction 220D-F2, was found to contain **23** and its derivatives [142]. This fraction displayed biofilm inhibitory activity against *S. aureus* and increased biofilms’ susceptibility to daptomycin and clindamycin. Using liquid chromatography coupled to UV detection and tandem mass spectrometry to identify the chemical contents of 220D-F2 revealed **23** (EA) and several ellagic acid derivatives (EADs) or sapogenin-related compounds [143]. A subsequent study reported that fraction 220D-F2 kills *Streptococcus pneumonia* planktonic cells and *pneumococcal* biofilms, and that 200 µg/mL of 220DF2 markedly decreased preformed pneumococcal biofilms. At the same time, a higher amount (i.e. 800 µg/mL of 220D-F2) was necessary to kill *S. pneumonia* biofilms formed on human pharyngeal cells. To identify the active component in 220D-F2, Fontaine et al. synthesized compounds **24** and **26** based on the molecular weight and ubiquity of rhamnose and xylose in plants [144]. It was hypothesized that antibiofilm activity associated with 220D-F2 is due to the 3-α-rha-EA **26** with the sugar moiety playing a pivotal role by making critical interactions with cellular components, thereby increasing anti-biofilm activity.

Recent studies reported the synthesis of ellagic acid carbohydrate derivatives **24**–**26** through a copper-mediated Ullmann coupling followed by a phenolic *O*-glycosylation [145]. The ellagic acid glycosides **24** and **25** displayed antibiofilm properties at concentrations of 512 µg/mL against *Streptococcus agalactaie* (Group B *Streptococcus*, GBS). The mechanism of action of ellagic acid glycosides remains mostly unknown, but scanning electron microscopy (SEM) has revealed that it limits initial adhesion in biofilm formation in GBS, and the biofilm to biomass ratios indicate that **24** and **25** impact biofilm formation but not planktonic cell growth.

## 5. In Silico Discovery

**Figure 4:**
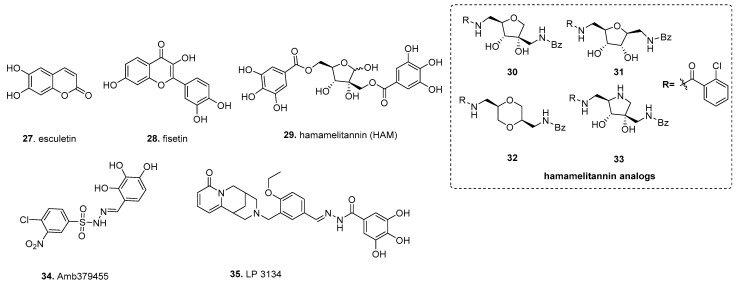
In silico-based discovery of biofilm inhibitors.

In silico-based studies are being increasingly utilized to identify biofilm inhibitors. By nature, this allows for the screening of extensive libraries without initial wet lab work. There are multiple approaches to in silico analysis, such as scaffold screens, analog development, and docking studies, which affords a degree of customization when employing computational screening techniques while offering opportunities to bias the screen towards particular targets [146] (Figure 4). 

### 5.1. Fisetin (from Ellagic Acid)

Building upon direct testing of gallic acid derivatives, in silico structure-based virtual screening using ellagic acid **23**, the initial scaffold discovered more potent biofilm inhibitors with broad species activity. Using multigenerational screening with a MAACC fingerprint database of the 57,346 member Chinese Natural Product Database, esculetin (**27**) was identified in the first round of screening, and fisetin (**28**), a plant flavonol from the flavonoid group of polyphenols, was identified in the second round (Figure 4) [147]. Both compounds inhibited biofilm formation of *S. aureus*; however, **28** had a substantially lower inhibitory dose, inhibiting biofilm formation by 90% at 16 µg/mL, whereas esculetin reduced biofilm formation by 77% with 128 µg/mL. The concentrations tested were deemed as being well below antibacterial levels. Additionally, **27** only exhibited inhibitory activity against *S. aureus* by inhibiting biofilm maturation, whereas **28** inhibited biofilm formation for *S. aureus* and multiple strains of *Streptococcus dysgalactiae* while also impacting biofilm initiation and development. With this approach, one can utilize a well-established inhibitor as a scaffold, and through multiple generations of in silico screening can enrich for potent biofilm inhibitors.

### 5.2. Hamamelitannin

In silico-based studies can be used to identify analogs of substrates for bacterial systems, allowing for the discovery of competitive inhibitors. An example of this approach is hamamelitannin (**29**), which targets the *S. aureus* RAP/TRAP quorum-sensing (QS) system (Figure 4). This QS system uses the secreted autoinducer peptide (AIP) “RNAIII-activating protein” (RAP) to induce phosphorylation of the “target of RAP” (TRAP) to impact gene expression as a function of cell density [148]. Interestingly, non-self AIPs have shown the ability to inhibit quorum sensing through the RAP/TRAP system by antagonizing RAP binding to TRAP [148]. Based on this, Kiran et al. sought to discover RIP analogs to inhibit biofilm formation using the ribosomal protein L2, an ortholog of TRAP, to develop a model of RIP, which was then used to screen 300,000 compounds from the available chemicals database (ACD) [149]. From this, **29** was identified as a non-peptide analog of RIP that exhibited no growth impact on *S. aureus*, but could compete against RAP, reducing surface attachment and virulence in the rat device-associated biofilm model. A later study found that **29** increases *S. aureus* biofilm susceptibility to antibiotics through agr and the traP QS system [150]. This was attributed to a decrease in cell wall thickness and a decrease in eDNA release through inhibition of TraP, resulting in inhibition of antibiotic-associated virulence and in vivo virulence of *C. elegans* and the mouse mammary glands infection model. Detailed medicinal chemistry efforts around the HAM scaffold found that analog 30 modulates quorum sensing as a potential antibiotic against *S. aureus* without showing cytotoxicity [151]. The EC_50_ of **30** is 0.389 µM, an approximately 400-fold improvement compared to **29** (EC_50_ = 145.5 µm). The key structural difference between the **29** and **30** includes the central tetrahydrofuran core and benzamides replace the labile ester. Follow-up studies by the same group centered around designing different hamamelitannin analogs by using ligand-based virtual screening where they modified only the hamamelose central core to different heterocyclic cores [152]. The 2,5-anhydro-D-allitol **31** and dioxane-derived **32** showed similar activity to that of lead analog **30** in the combination treatment setup. Finally, the pyrrolidine-derived analog **33** with different N-alkylation was found to be completely inactive in inhibiting *S. aureus* biofilm formation [153].

### 5.3. Amb379455

Another option for screening is through in silico docking of compounds into the three-dimensional structure of the intended target, which allows screening extensive virtual libraries for compounds that likely can bind with specificity [154]. In one study, a diguanylate cyclase was used as a target protein. Fernicola et al. created a 14-point pharmacophore hypothesis of *Caulobacter crescentus*’s PleD, representing one of the first DGCs whose crystal structure is known. It is a common template used for DGC-based in silico docking, in complex with a GTP analog to define docking parameters to identify competitive antagonists for the active site [155,156]. A 3D representation of these docking parameters was presented in Figure 1 of Fernicola et al. This led to the identification of Amb379455 (**34**), a pyrogallol unit containing a sulfonohydrazide functional group, as a potential inhibitor from the 2.3 × 10^7^ member ZINC database (Figure 4). The close structural analysis with other analogs suggests that phenols and nitro position is crucial for the activity. As this in silico screen utilized a known DGC, the Fernicola et al. investigated whether Amb379455 **34** can specifically impact PleD. Not only was Amb379455 able to inhibit PleD with a 50% enzymatic inhibitory concentration (IC_50_) of 11.1 ± 1.1 µM, in a dose-dependant manner, it inhibited the *P. aeruginosa* DGCs WspR and YfiN, which are critical in c-di-GMP signaling and biofilm formation [157,158]. As YfiN lacks an inhibitory site (I-sites), a common feature among DGCs needed for allosteric inhibition of the DGC via the substrate c-di-GMP, the mechanism of inhibition for **34** is likely specific for the active site of these DGCs. Based on the pharmacophore hypothesis, **34** likely targets the active sites of both PleD and YfiN; the phenolic OH, which coordinates with magnesium, whereas sulphonamide, nitro functional groups interact with N335 and R366 residue, respectively. This notion is supported by the fact that **34** can competitively displace MANT-GTP from YfiN. This example illustrates that in silico discovery can achieve large gains in structural knowledge in tandem with inhibitor discovery, making this a valuable discovery tool. 

### 5.4. LP 3134 

Another study used the same target, *C. crescentus*’s PleD, in complex with c-di-GMP, to identify an active site’s inhibitor [159]. Sambanthamoorthy et al. used the active site and residues with GMP interactions to screen 15,000 compounds from a guanine/oroidin moiety-focused library which are illustrated in their Figure 1. Lead compounds were further refined with MOE software based on electrostatic energy, binding restrictions, and energy minimization to generate the final PleD-compound complexes. Four compounds were found to decrease biofilm formation, without impacting cell growth, in *P. aeruginosa* and *A. baumannii* using both crystal violet staining and confocal microscopy, but only LP-3134 (**35**), a heterocyclic compound containing N-benzylidenebenzohydrazide and pyridine-2 one fused with bicyclic amine moiety, was able to disperse intact biofilms for both *P. aeruginosa* and A. *baumanii*. (Figure 4) Based on the in silico complex of **35** and PleD, three of the four GMP-PleD hydrogen bonds are satisfied by the phenolic OH from N-benzylidenebenzohydrazide form. The fourth hydrogen occurs between the singular oxygen of the pyridine-2 fused ring and the amide backbone of R366. Additionally, **35** was found to inhibit surface attachment for *P. aeruginosa* and prevent biofilm formation on silicone catheters. Sambanthamoorthy et al. also reported that **35** had an IC_50_ of 44.9 µM against the *P. aeruginosa* DGC WpsR. 

## 6. Therapeutic Drug Conjugates

Beyond the discovery of novel biofilm inhibitors, there has been increasing interest in the advances of therapeutic drug conjugates for cancer and drug delivery and utilizing that class of therapeutics to target biofilms [160,161]. Here, antimicrobials can be linked to antibodies, peptides, or various compounds to improve (i) target specificity, (ii) penetration of the biofilm matrix, or (iii) condition-specific activity [162,163,164,165,166,167,168]. This approach can reduce the efficacy of biofilms’ protective and resistant nature when challenged by traditional antibiotics and has found success in recent studies and clinical trials [169,170,171].

**Figure 5:**
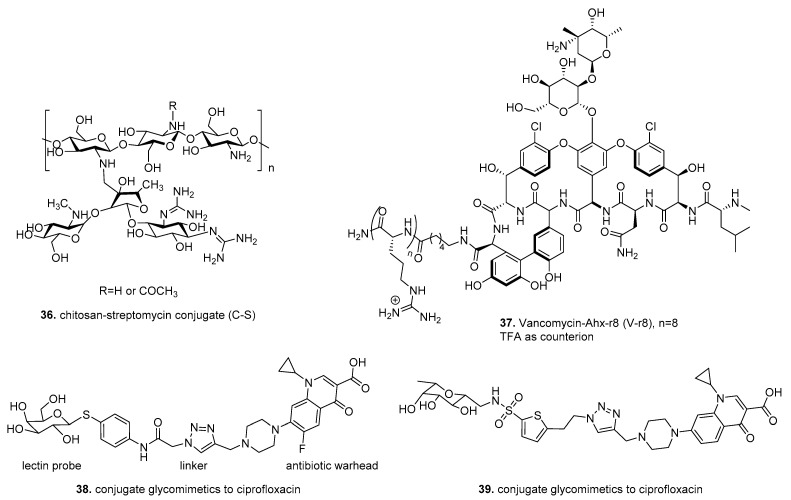
Therapeutic drug conjugates.

An early example of therapeutic drug conjugates to target biofilms utilized chitosan, the N-deacetylated derivative of chitin, as a delivery method for the aminoglycoside antibiotic streptomycin to target cell membranes and penetrate preformed biofilms [172,173]. (Figure 5) The conjugate **36** was synthesized by the reduction of Schiff base formed by the reaction of aldehyde from streptomycin and amine from chitosan. Dose-dependent analysis indicated that the C-S conjugate **36** (at 0.125, 0.25, and 0.5 mg/mL) was more efficient in disruption of *L. monocytogenes* biofilms than chitosan, streptomycin, or a mixture of both. The anti-biofilm efficacy of C- S conjugate **36** was effective against Gram-positive organisms (*L. innocua*, *L. welshimeri*, *E. faecalis* and *S. aureus*) but not Gram-negative organisms (*P. aeruginosa*, *S. typhimurium*).

Another study designed to improve antibiotic penetration into biofilms conjugated the antibiotic vancomycin to a molecular transporter (MoTr), which has been previously shown to penetrate cell membranes [169,174]. Antonoplis et al. utilized the guanidinium-rich MoTr D-octoarginine, which has 30-fold higher MIC than vancomycin for planktonic cells, and alone was ineffective against biofilm grown cells. The standard EDC condition used for coupling of unprotected NH2- Ahx−r8 to the C terminus of vancomycin to produce V−r8 (**37**) (Figure 5). Vancomycin-octoarginine (V-r8) was markedly more effective at eradicating methicillin-sensitive and methicillin-resistant *S. aureus* biofilm-grown cells compared to unconjugated vancomycin. However, V-r8 did not improve vancomycin’s ability to kill planktonic cells suggesting that V-r8 primarily aids in biofilm penetration, not cell penetration. Additional studies using *S. aureus* biofilms showed that **37** killed 91.6% of biofilm-associated cells, while unconjugated vancomycin resulted in only 35% killing. In a murine wound infection model, **37** eliminated 97% of biofilm-associated MRSA without acute dermal toxicity.

In contrast to the previous drug conjugates, which improved cell and biofilm penetration, Meiers et al. explored the conjugation of ciprofloxacin to glycomimetics to target LecA LecB, extracellular biofilm-related virulence factors of *P. aeruginosa* [175]. This strategy targets ciprofloxacin delivery to the infection site, reducing off-target effects and gaining antibiotic resistance. Sulfonamide-capped mannosides, and C-glycosides combining pharmacophores of its natural ligands, fucose, and mannose showed excellent binding affinity against LecB and inhibited in vitro biofilm formation [176].

Drug conjugates **38**, **39** were synthesized using copper-catalyzed [3 + 2] cycloaddition of terminal azides from lectin probe and terminal alkyene from the antibiotic ciprofloxacin. (Figure 5) Utilizing *P. aeruginosa* biofilms formed on pegs, the two lectin-targeting conjugates (**38** and **39**) were compared with unconjugated ciprofloxacin at 100 μM. The lectin-targeted conjugates **38**, **39** reached higher concentrations in the bacterial biofilm than the unmodified ciprofloxacin when analyzed with LC/MS. In vitro, ADMET data, which determine pharmacological properties of compounds, showed **38**, **39** conjugates are metabolically stable and showed no cytotoxicity at 100 μM after 48 h incubation against a human embryonic kidney cell line (HEK 293) and adenocarcinoma human alveolar basal epithelial cells (A549). 

Collectively, these studies revealed that therapeutic drug conjugates present desirable therapeutic options utilizing a potent antimicrobial warhead with a higher specific delivery method.

## 7. Conclusions

Biofilm formation is a beneficial growth mode that protects microorganisms from antimicrobial agents and immune responses, making biofilm-associated cells challenging to eradicate. Thus, there is a need for biofilm inhibitors that can render these bacteria susceptible to treatment strategies and removal. The discovery of novel inhibitors is an essential step in reaching this goal, and numerous approaches have proven successful. This review covered the two major approaches to inhibitor discovery, screening-based and in silico, and discussed structural information obtained through SAR strategies. We have provided examples of high-throughput screening strategies using "whole-cell" and targeted approaches that identified cahuitamycins A-E, auromomycin, skyllamycins A-C, and terrein.

Additionally, we discussed targeted/pathway screens, which discovered ebselen, DI-3, AA-861, parthenolide, and ellagic acid. Furthermore, we examined three distinct in silico discovery methods that identified fisetin, hamamelitannin, Amb379455, and LP 3134 as biofilm inhibitors. Finally, we explored the realm of drug conjugates that utilize antibiotics linked to compounds that aid in cell/biofilm penetration and target specificity such as chitosan-streptomycin, V-r8, and the glycomimetic-ciprofloxacin conjugates. Though not expansive, these examples each present a unique approach to compound discovery with particular advantages, methods for inhibitor characterization, and current SAR knowledge. As our understanding of mechanisms of biofilm formation improves, we anticipate seeing an expansion of biofilm inhibitor studies beyond the typical target of quorum sensing and into other critical biofilm regulatory systems such as c-di-GMP signaling. Similarly, mechanistic understanding of biofilm formation will aid in the refinement and development of in silico-based and therapeutic drug conjugate studies. Finally, the integration of in vitro model systems of biofilm-associated diseases, such as organoid models, in the development of screening platforms will lead to the identification of novel biofilm inhibitors.

## Figures and Tables

**Table 1:** Biofilm inhibitors discussed, their target pathogen, discovery method, and inhibitory activity. This includes biofilm inhibitory concentration (BIC50), enzymatic inhibitory concentration (IC50), biofilm dispersal concentration (BDC50), minimal biofilm eradication concentration (MBEC50), percent reduction in biofilm biomass (biofilm reduction), and percent inhibition of enzyme activity (enzyme inhibition).

Compound Name	Target Organism	Discovery Method	BIC_50_/ IC_50_	BDC_50_/ MBEC_50_	Biofilm Reduction	Enzyme Inhibition
Cahuitamycin C (**3**)	*A. baumannii*	Cell Based HTS	14.5 μM	692 μM	-	-
Cahuitamycin D (**4**)	*A. baumannii*	Mutasynthetic Studies	8.4 μM	535 μM	-	-
Cahuitamycin E (**5**)	*A. baumannii*	Mutasynthetic Studies	10.5 μM	-	-	-
Auromomycin (**6**)	*V. cholerae*	Cell Based HTS	60.1 μM	-	-	-
Derivative 25 (**13**)	*V. cholerae*	SAR Studies	6.0 μM	13 μM	-	-
Skyllamycin A (**14**)	*P. aeruginosa*	Cell Based HTS	>250 μM	-	-	-
Skyllamycin B (**15**)	*P. aeruginosa*	Cell Based HTS	30 μM	-	-	-
Skyllamycin C (**16**)	*P. aeruginosa*	Cell Based HTS	60 μM	-	-	-
Terrein (**17**)	*P. aeruginosa*	Cell Based HTS	-	-	-	81.10%
Ebselen (**18**)	*P. aeruginosa*	In vitro HTS	-	-	-	80–90%
DI-3 (**20**)	*V. cholerae*	Cell Based HTS	1.0 μM	-	-	-
AA-861 (**21**)	*E. coli*	Phenotypic screen	-	-	Near 40%	-
Parthenolide (**22**)	*E. coli*	Phenotypic screen	-	-	Near 40%	-
Ellagic acid (**23**)	*S. aureus*	Targeted screening	50 μM	-	50%	-
3-β-xyl-EA (**24**)	*S. aureus*	SAR Studies	512 μg/mL	-	-	-
3-α-ara-EA (**25**)	*S. aureus*	SAR Studies	512 μg/mL	-	-	-
Fiscetin (**28**)	*S. aureus*	Structure Based In silico Screen	-	-	90%	-
Hamamelitannin (**29**)	*S. aureus*	Structure Based In silico Screen	145.5 μM	-	-	-
Derivative 38 (**30**)	*S. aureus*	SAR Studies	0.389 μM	-	-	-
Amb379455 (**34**)	*C. crescentus*	In silico Docking	11.1 μM	-	-	-
LP3134 (**35**)	*C. crescentus*	In silico Docking	44.9 μM	-	-	-
V-r8 (**37**)	*S. aureus*	Drug-conjugation	-	9.5–20 μM	-	-
Congujate 7b (**39**)	*P. aeruginosa*	Drug-conjugation	-	-	80–90%	-

## Abbreviations

ADMET	Absorption, distribution, metabolism, and excretion properties and toxicities which are useful in evaluating pharmecuetical properties of prospective therapeutics.
Antibacterial	A compound that kills bacteria or prevents bacterial growth.
BDC_50_	The concentration at which 50% of the preformed biofilm is dispersed.
BIC_50_	The concentration at which 50% of biofilm formation is inhibited.
Biofilm inhibitor	A compound that inhibits or negatively impacts biofilm formation. This includes the prevention of biofilm formation as well as dispersal and disruption of preformed biofilms.
DGC	Diguanylate cyclase
EC_50_	The concentration at which treatment is 50% effective.
EDC condition	1-Ethyl-3-(3-dimethylaminopropyl)carbodiimide (EDC) is used as a carboxyl activating agent to form amide bonds in coupling reactions.
IC_50_	The concentration at which enzymatic activity is 50% inhibited.
MBEC	Minimal biofilm eradication concentration.
MIC	Minimal inhibitory concentration.
MoTr	Cell associated or cell-penetrating molecular transporter.
PDE	Phosphodiesterase
QS	Quorum sensing
